# Comparison of the Antioxidant Activities and Phenolic Content of Five *Lonicera* Flowers by HPLC-DAD/MS-DPPH and Chemometrics

**DOI:** 10.1155/2020/2348903

**Published:** 2020-04-01

**Authors:** Rong-rong Zhou, Xue-hui Liu, Lin Chen, Jian-hua Huang, Xue-juan Liang, Dan Wan, Shui-han Zhang, Lu-qi Huang

**Affiliations:** ^1^Changchun University of Chinese Medicine, College of Pharmacy, Changchun, China; ^2^Hunan Academy of Chinese Medicine, Research Institute of Chinese Medicine, Changsha, China; ^3^China Academy of Chinese Medical Sciences, National Resource Center for Chinese Materia Medica, Beijing, China

## Abstract

The *Lonicera* plants (family Caprifoliaceae) with strong antioxidant activity are used as potential health-supporting phytochemicals. Studying the detailed relationships between bioactive compounds and their antioxidant activity is important for further comprehensive development and application of them. In this paper, the antioxidant capacities and compositions of five species of *Lonicera* flowers were investigated by using the online HPLC-DAD/MS-DPPH method. Results indicated that the samples contained higher amounts of phenols had better antioxidant activity. Furthermore, principal component analysis and linear regression were further used to analyze the correlations between antioxidant capacity and compounds and find the compounds having higher contribution to antioxidant activity. 5-O-Caffeoylquinic acid, 4-O-caffeoylshikimic acid, methyl-5-O-caffeoylquinate, 1,4-di-O-caffeoylquinic acid, and 3,4,5-tri-O-caffeoylquinic acid were screened as stronger antioxidant candidates. In this study, HPLC-DAD/MS and antioxidant activity methods were combined together to analyze the compounds' information and activity assays of *Lonicera*, which might provide more evidence for its quality control.

## 1. Introduction

Epidemiological studies strongly showed antioxidant protection effects against various diseases or illness relevant to oxidative stress, such as aging, cardiovascular diseases, cancer, stoke, diabetes, Alzheimer's disease, hypertension, and inflammation [1–5]. Various antioxidants from natural products are excellent candidates, e.g., phenolic acids, tannins, lignans, and flavonoids) [6–10]. Thus, searching antioxidants from natural products has attracted a lot of attention.

*Lonicera* is one of the most crucial genera in the Caprifoliaceae family [11–13]. In China, there are several *Lonicera* species which have been used as herbal to treat headache, pharyngodynia, acute fever, respiratory infection, epidemic disease, and pyocutaneous disease [14–18] for centuries. Recently, a lot of studies have disclosed the existence of phenolic acids, flavonoids, saponins, and iridoids in *Lonicera* species [19–23]. In the Chinese Pharmacopoeia (2015), four species, including *L. hypoglauca, L. confusa, L. fulvotomentosa and L. macranthoides*, were recorded as Flos Lonicerae (Shanyinhua in Chinese). Antioxidant activity was the shared pharmacological activity of *Lonicera* species.

Some studies were proposed to compare or discover these antioxidants, namely Li et al., found that *Lonicera macranthoides* presented better antioxidant activities than *Lonicerae japonicae* [24]. However, there is a lack of systematic investigations on the correlations between bioactive components and antioxidant capacity of Flos Lonicerae. Moreover, the antioxidative capacities of different varieties of Flos Lonicerae were usually affected by the contents of antioxidant compounds and their oxidation resistances. Analysis of antioxidant capacities of different varieties of Flos Lonicerae cannot be performed accurately by any single antioxidant compound. Accordingly, the content and oxidation resistance of different antioxidant components should be considered together to comprehensively evaluate the antioxidant activities of Flos Lonicerae.

Hyphenated techniques are needed for the analysis of these antioxidants from complex samples. “HPLC-DPPH method” is one of these kinds of hyphenated techniques and had been widely applied to identify antioxidants in a complex matrix, such as herbs and natural products [25–30]. In the current study, an online HPLC technique coupled with MS^n^ and the DPPH-HPLC method was used to screen and characterize the antioxidants in Flos Lonicerae. Firstly, the antioxidant activities of five *Lonicera* flowers were estimated. Then, a DPPH-HPLC assay was used to screen the active compounds, and LC-MS/MS was further used to identify the active compounds. Furthermore, the correlations between bioactive compounds and their antioxidant capacity were also constructed by using a linear regression model.

## 2. Experimental

### 2.1. Apparatus

Agilent 6530 Accurate-Mass Q-TOF-LC/MS system coupled with a Quat-Pump was utilized in this study. An InertSustain-C18 (5-micron, 4.6 × 250 mm) column was used to separate the peaks in samples. The mobile phases A (0.1% formic acid in water) and B (acetonitrile) were programmed as follows: 0∼5 min, 5% B; 5∼10 min, 5%∼23% B; 10∼15 min, 23% B; 15∼20 min, 23%∼36% B; 20∼25 min, 36% B; 25∼45 min, 36%∼50% B; 45∼55 min, 50%∼70% B; 55∼60 min, 70%∼72% B. The injection volume was 10 μL, and the flow rate was 1 mL/min and column temperature was 28°C. The LC-MS system was controlled by an Agilent ChemStation to record total ion chromatograms and mass spectra, with following conditions: dry gas: N2, 8.0 L/min, 322°C; sheath gas: N2, 11 L/min, 322°C; fragmentor voltage, 180 V; capillary voltage, 3500 V).

### 2.2. Reagents

DPPH was purchased from Sigma-Aldrich (Steinheim, Germany). Analytical grade ethanol, methanol, and formic acid were purchased from Sinopharm Chemical Reagent Co., Ltd (China). The chromatographic grade of acetonitrile was purchased from Merk (Germany). Ultrapure water was purified and filtered by a Milli-Q water system (Millipore, Bedford, MA, USA).

### 2.3. Preparation of *Lonicera*

Thirty batches of flower buds of *Lonicera* species (6 batches of each species) were collected from cultivated markets in China. The species of all samples were authenticated by Prof. Zhao-ming Xie (Research Institute of Chinese Medicine, Hunan Academy of Chinese Medicine, Changsha, Hunan). All samples were powdered and screened through 80 mesh sieves. 3.0 g prepared powder of each sample was accurately weighed, mixed with 30 mL of 80% ethanol, and then extracted by ultrasonic extraction for 40 min. After adjustment to the initial weight by ethanol, the supernatant was collected and filtered through a 0.45 *μ*m membrane filter before using for HPLC analysis.

### 2.4. DPPH Assay for Evaluation of Antioxidant Activity

Analysis of antioxidant activity was followed by a previous method with brief improvement [31]. The DPPH· solution was used as the control. The ability to scavenge DPPH· was calculated according to the following equation: [(*A*_control_ − *A*_sample_)/*A*_control_] × 100%. IC_50_ (50% inhibition) was calculated based on the graph plotting inhibition percentage. The above experiment results were expressed as the mean ± standard deviation (SD), parallel tested three times.

### 2.5. HPLC-DAD/MS-DPPH Assay

Prepared samples' extract (300 *μ*L) was mixed with methanol to 1.5 mL and then reacted with DPPH· (300 *μ*L, 30 mg/mL) at 38°C for 40 min. Then, each sample was passed through a 0.22 *μ*m filter before HPLC-DAD/MS analysis. Sample 1.5 mL without DPPH· was used as the control. MS data were acquired across the range *m/z* 50–1000 in negative ion modes with an acquisition rate of 1.02 spectra/s, by the Agilent 6530 Accurate-Mass QTOF/MS system with an ESI interface.

### 2.6. Statistical Analysis

Averaged result of three replicates for each sample was used for the subsequent statistical analysis. The principal component analysis (PCA) method was used to transform the original measured variables into new uncorrelated variables (principal components) and to display the relationship among samples or different species. Also, the linear regression model was proposed by using the software Matlab.

## 3. Results and Discussion

### 3.1. Antioxidant Activity of Five *Lonicera* Flowers

The antioxidant activities of these five species are shown in Table 1. The species *L. macranthoides* showed excellent capacity to scavenge the DPPH radical with the IC_50_ value of 235.27 *μ*g/ml (Table 1). The results indicated that *L. macranthoides* flowers have strong antioxidant activity. Therefore, HPLC-DAD/MS-DPPH was further applied to screen and characterize contained antioxidants.

### 3.2. Screening and Analyzing Antioxidants by HPLC-DAD/MS-DPPH

**Figure 1:**
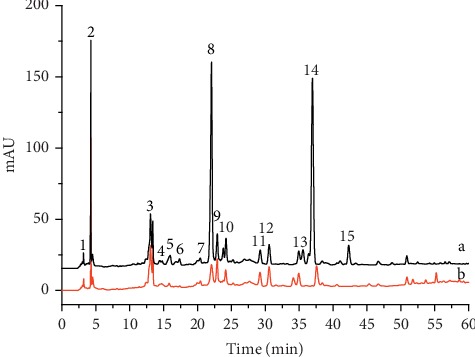
HPLC (a) and HPLC-DPPH (b) of *Lonicera japonica* monitored by HPLC-DAD-QTOF-MS/MS in the negative ion mode.

HPLC-DAD/MS-DPPH was a rapid method and can screen antioxidants from complex mixtures without complicated sample pretreatment. It was believed that the peak areas of these antioxidants in the HPLC chromatogram will be decreased if they react with DPPH•.  Figure 1 shows the results of *Lonicera japonica* before and after reaction with DPPH•. It can be found that the peak areas of compounds 1 to compound 15 were obviously decreased after reacting with the DPPH• solution. Therefore, these compounds (compound 1 to compound 15) were considered to have antioxidant properties.

Then, both positive and negative ion modes were determined to obtain the appropriate ionization of these compounds. Peak identification was assigned primarily by means of the fragment ions and corresponding MS^2^ fragment ions. The collision energy for MS^2^ experiments was optimized in the range from 15 to 60 eV, and the best results for structure identification were obtained at 35 eV.

Caffeoylquinic acids were reported as main metabolites in *Lonicera* species in previous studies, and their accurate identification was a very difficult task due to their similar regional and geometrical isomerism. The distinct MS/MS fragmentation pathways and elution order could be used to assist in the structure identification (Table 2). Four compounds (1–4) were pairs of isomers with molecular weight at *m/z* value of about 353.08 (C_16_H_18_O_9_). By analysis of the MS/MS spectra and comparison with standards, 1, 2 and 3 were confirmed as 3-O-caffeoylquinic acid, 5-O-caffeoylquinic acid, and 4-O-caffeoylquinic acid. Then, we speculated that compound 4 could be cis-4-O-caffeoylquinic acid. It has been reported that caffeoylquinic acid derivatives underwent trans-cis isomerization after being exposed to UV, and cis caffeoylquinic acids had been found in the natural resource. Then, we radiated 4-O-caffeoylquinic acid with UV light at 254 nm for 65 min, using a similar approach and by comparison with standards, compounds 11–15 were assigned as 3,4-di-O-caffeoylquinic acid, 3,5-di-O-caffeoylquinicacid, 4,5-di-O-caffeoylquinic acid, 1,4-di-O-caffeoylquinic acid, and 3,4,5-tri-O-caffeoylquinic acid, respectively. Thus, combination of HPLC-DAD/MS techniques with DPPH-HPLC to detect antioxidant compounds has the advantages of that chromatography as the separation method, an ESI-MS/MS as the identification method, and a DPPH assay as the activity evaluation method.

### 3.3. Principal Component Analysis of Compound Content and Antioxidant Activity

**Figure 2:**
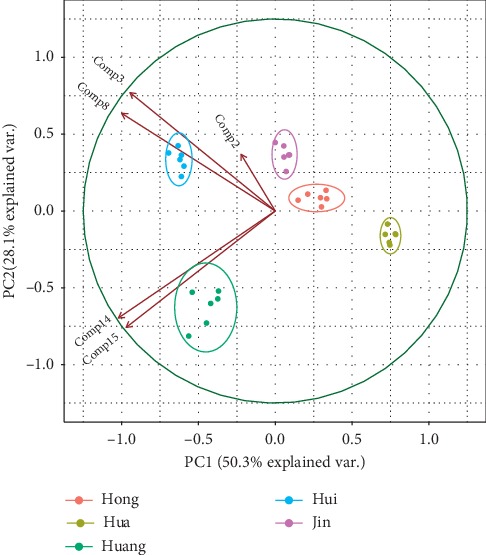
Score and loading plot from the PCA of main antioxidant compounds.

PCA could convert variables into a few comprehensive principal components to exhibit the relationships of data. The peak areas of these 16 compounds selected by HPLC-DAD/MS and DPPH-HPLC were imputed to PCA for data analysis. The first two principal components (PC1 and PC2) were found and accounted for approximately 81.7% of total variances, indicating that PC1 and PC2 contained most information of all variables. Generally, the score plot could discriminate differences in samples as similar or nonsimilar. As shown in Figure 2, *L. japonica*, *L. macranthoides*, *L. fulvotomentosa*, *L. confuse*, and *L. hypoglauca* could be clearly discriminated from each other. The loading values for these compounds could be used to estimate the importance or contributions to the classification. As can be seen from Figure 2, five compounds, com 2, 3, 8, 14, and 15, had the higher loading values in the classification of different species.

**Figure 3:**
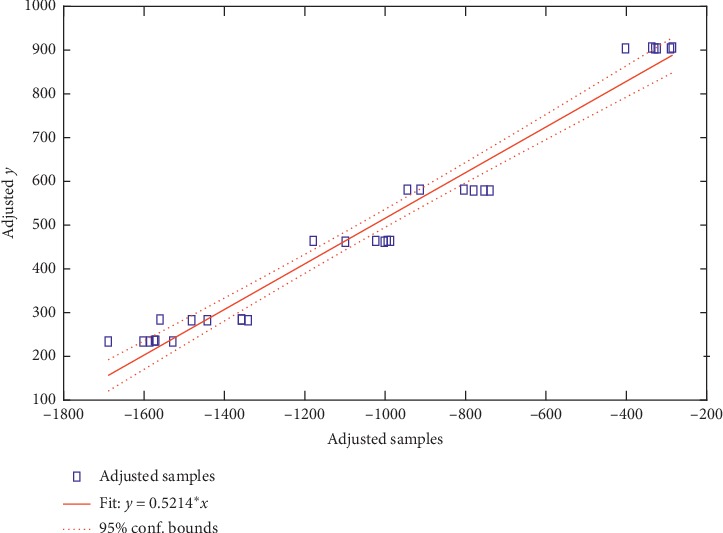
The fitted model based on selected five compounds and antioxidant activity.

Furthermore, a linear regression model was carried out to identify relationships between these five compounds and the samples' antioxidant activity in Flos Lonicerae. In the linear regression model establish process, the peak areas of these five selected compounds were used as the independent variables (*x*), and the IC_50_ values obtained by the DPPH· assay method were used as the dependent variables (*y*). A linear regression model was established based on the independent variables (*x*) and the dependent variables (*y*). The linear model is listed in Figure 3. Adjusted data points are computed by adding the residual to the adjusted fitted value for each observation. Two indexes, *R*-squared and adjusted *R*-squared, were calculated to estimate the linear regression model. In the current study, the peak areas of com 2, 3, 8, 14, and 15 were fitted with their antioxidant activities. The *R*-squared and adjusted *R*-squared values were 0.966 and 0.959, respectively. These results showed that these five compounds are in strong relation with their antioxidant activities.

## 4. Conclusions

In this paper, the antioxidant assays demonstrated that all the samples exerted perfect antioxidant capacity in the following order: *Lonicera macranthoides* > *Lonicera fulvotomentosa* > *Lonicera japonica* Thunb > *Lonicera hypoglauca* Miq > *Lonicera confusa*. In addition, the results from PCA and DPPH-spiking HPLC analysis confirmed that 5-O-caffeoylquinic acid, 4-O-caffeoylshikimic acid, methyl-5-O-caffeoylquinate, 1,4-di-O-caffeoylquinic acid, and 3,4,5-tri-O-caffeoylquinic acid contributed to antioxidant activity. Therefore, the HPLC-DAD/MS-DPPH analysis method is expected to provide efficient, time-saving, and sensitive technology for screening and identification of radical scavengers from a complex matrix, which will benefit for the further utilization of Flos Lonicerae.

## Figures and Tables

**Table 1:** Antioxidant activities of five *Lonicera* flowers in the DPPH assay.

Samples	DPPH (IC_50_, *μ*g/ml)
*Lonicera macranthoides* Hand.-Mazz.	235.27 ± 1.21^*∗*^
*Lonicera fulvotomentosa* Hsu et S. C. Cheng	284.87 ± 1.05^*∗*^
*Lonicera japonica* Thunb.	464.48 ± 1.32
*Lonicera hypoglauca* Miq.	580.96 ± 0.95
*Lonicera confusa* (Sweet) DC.	905.23 ± 1.02

Each value is mean ± SD (*n* = 3).

**Table 2:** ESI-MS^2^ results of antioxidants in *Lonicera*.

No.	tR/min	[M–H]– (*m/z*)	Exptl. exact mass	Fragment ions (*m/z*)	Error (ppm)	Molecular formula	Identification
1	4.281	[M-H]-	353.0877	191.0559, 179.0343	0.53	C16H18O9	3-O-Caffeoylquinic acid
2	15.983	[M-H]-	353.0875	191.0557	−2.57	C16H18O9	5-O-Caffeoylquinic acid
3	19.586	[M-H]-	335.0773	179.0346, 161.0242, 135.0446, 317.0667	0.82	C16H18O9	4-O-Caffeoylshikimic acid
4	19.754	[M-H]-	353.0877	173.0453, 179.0345, 191.0558	0.45	C16H18O9	cis−4-O-Caffeoylquinic acid
5	19.987	[M-H]-	367.1026	161.0244, 135.0452	2.37	C17H20O9	Methyl-4-O-caffeoylquinate
6	21.271	[M-H]-	404.3684	371.0243, 179.0455	−2.15	C17H24O11	Secoxyloganin
7	21.623	[M-H]-	335.0775	179.0344, 135.0451	1.85	C16H16O8	3-O-Caffeoylshikimic acid
8	23.290	[M-H]-	367.1026	179.0345, 135.0452, 191.0559, 161.0243	0.64	C17H20O9	Methyl-5-O-caffeoylquinate
9	27.345	[M-H]-	381.1084	161.0243, 135.0452	−2.35	C18H22O9	Ethyl-4-O-caffeoylquinate
10	30.296	[M-H]-	381.1081	179.0343, 135.0454, 191.0558, 161.0245	−2.17	C18H22O9	Ethyl-5-O-caffeoylquinate
11	33.269	[M-H]-	515.1193	353.0876, 173.0453, 179.03445, 191.0559	−0.82	C25H23O12	3,4-di-O-Caffeoylquinic acid
12	35.504	[M-H]-	515.1196	353.0876, 191.0558, 179.0345	0.69	C25H23O12	3,5-di-O-Caffeoylquinic acid
13	37.875	[M-H]-	515.1195	353.0874, 173.0452, 179.0345, 191.0559	−3.12	C25H23O12	4,5-di-O-Caffeoylquinic acid
14	42.663	[M-H]-	515.1197	353.0876, 173.0453, 191.0562, 179.0347	−2.53	C25H23O12	1,4-di-O-Caffeoylquinic acid
15	43.397	[M-H]-	677.1592	515.1193, 353.0876	−0.49	C34H30O15	3,4,5-tri-O-Caffeoylquinic acid

## Data Availability

The data used to support the findings of this study are available from the corresponding author upon request.
